# *SLCO1B1* c.388A>G Polymorphism Is Associated with HDL-C Levels in Response to Atorvastatin in Chilean Individuals

**DOI:** 10.3390/ijms160920609

**Published:** 2015-08-31

**Authors:** Yalena Prado, Nicolás Saavedra, Tomás Zambrano, Jenny Lagos, Alexy Rosales, Luis A. Salazar

**Affiliations:** Center of Molecular Biology and Pharmacogenetics, Department of Basic Sciences, Scientific and Technological Bioresource Nucleus (BIOREN), Universidad de La Frontera, Temuco 4811230, Chile; E-Mails: yalenapradovizcaino@gmail.com (Y.P.); nsaavedracuevas@gmail.com (N.S.); t.andreszambrano@gmail.com (T.Z.); tm.jenny.lagos@gmail.com (J.L.); tm.arosales@gmail.com (A.R.)

**Keywords:** *SLCO1B1*, OATP1B1, gene polymorphisms, lipid-lowering therapy, hypercholesterolemia, atorvastatin, pharmacogenetics

## Abstract

The use of statins as the preferred lipid-lowering therapy has clearly demonstrated its efficacy in the treatment of hypercholesterolemia, reducing also the risk of coronary events and cardiovascular disease mortality. In this study, we assessed single nucleotide polymorphisms (SNPs) in the *SLCO1B1* gene and their effect on atorvastatin response. We included 129 Chilean hypercholesterolemic patients undergoing 10 mg/day of atorvastatin therapy during 4 weeks. Lipid profile was determined before and after drug administration. Genotyping of *SLCO1B1* rs4149056 (c.521T>C) SNP was performed with allele-specific polymerase chain reaction, whilst polymerase chain reaction-restriction fragment length polymorphism (PCR-RFLP) was used for genotyping the *SLCO1B1* rs2306283 (c.388A>G) variant. After statin therapy, concentrations of TC, LDL-C and TG had a decrease from baseline (*p* < 0.05). Also, HDL-C levels increased (*p* < 0.05). Minor allele frequencies for the rs2306283 and rs4149056 variants were 0.547 and 0.136, respectively. LDL-C response to atorvastatin was not associated with the *SLCO1B1* rs4149056 nor the rs2306283 polymorphisms (*p* > 0.05). However, the latter SNP was associated with HDL-C variability after atorvastatin medication (*p* = 0.02). This study indicates that LDL-C reduction following atorvastatin therapy is not influenced by the SNPs evaluated. In addition, the polymorphism rs2306283 at the *SLCO1B1* gene determines greater HDL-C concentrations in response to atorvastatin medication in Chilean hypercholesterolemic subjects.

## 1. Introduction

Statins are competitive and reversible inhibitors of 3-hydroxy-3-methyl-glutaryl-CoA reductase (HMGCR), the rate-limiting enzyme for cholesterol synthesis. By this mechanism, statin medication effectively reduces intracellular cholesterol levels. In order to restore normal levels of sterols, the SREBP pathway is triggered [1], activating several genes such as the low-density lipoprotein receptor (*LDLR*), which in turn cause a compensatory increase in the expression of LDLRs in liver cells, enhancing plasmatic clearance of this lipoprotein and thus, reducing circulating low-density lipoprotein cholesterol (LDL-C) and improving other lipid parameters as well, such as triglycerides (TG) and high density lipoprotein (HDL) cholesterol (HDL-C) [2]. In addition, many studies report different pleiotropic effects of statins, such as anti-inflammatory, antioxidant, antithrombotic and neuroprotective activity. Also, there is evidence indicating an increase in nitric oxide expression levels, contributing with the prenylation of isoprenoids and modifying plaque stabilization and subsequent atherosclerosis risk [3]. At present, statins are considered to be highly effective in the treatment of hypercholesterolemia. However, there are a number of patients presenting severe adverse drug reactions (ADRs) such as liver function disorders, muscle myopathy and rhabdomyolysis [4,5]. Also, different studies indicate that clinical results using lipid-lowering therapy with statins hold great variability between patients, greatly limiting their beneficial effects [6,7]. Recent investigations have been focusing on genetic variations at hepatic efflux and influx transporters as the potential responsible for these differences, such as the *SLCO1B1* (solute carrier organic anion transporter family, member 1B1) gene, which encodes the organic anion-transporting polypeptide OATP1B1 [8], a protein of 691 amino acids, located on the basolateral membrane of hepatocytes and small intestinal enterocytes. OATP1B1 mediates the transport of a wide number of exogenous and endogenous substances in hepatocytes, including statins, e.g., atorvastatin, pravastatin, rosuvastatin and cerivastatin [9,10]. Of the many variations reported at the *SLCO1B1* locus, two single nucleotide polymorphisms (SNPs)—c.388A>G (rs2306283) and c.521T>C (rs4149056)—have been largely studied, mainly for affecting the function of the carrier and impairing the transport of statins [10,11,12]. The polymorphism c.388A>G has been associated with elevated activity of OATP1B1 and lower statin concentration in plasma [10,13]. In contrast, the OATP1B1 c.521T>C polymorphism is associated with a reduction in the activity of the transporter and therefore, increased plasma levels of statins [14]. Also, the haplotype designated as SLCO1B1*15, formed by both c.388A>G and c.521T>C, has been associated with decreased activity of the OATP1B1 transporter [15]. In relation to atorvastatin, other reports have also found an association between genetic polymorphisms at *SLCO1B1* and response to this drug [8,13]. Since atorvastatin is an OATP1B1 substrate, medication with this drug may be subjected to interindividual variability in hypercholesterolemic patients according to the genetic variants aforementioned. Hence, our goal was to evaluate the influence of the common c.388A>G and c.521T>C polymorphisms in the *SLCO1B1* gene on lipid-lowering response to low-dose atorvastatin in Chilean hypercholesterolemic patients.

## 2. Results

### 2.1. Demographics and Clinical Variables

Table 1 shows the clinical and demographic characteristics of the hypercholesterolemic patients studied. Atorvastatin treatment was well tolerated by all patients. No skeletal muscle abnormalities or other adverse reactions were detected after treatment. Concentrations of TC, LDL-C and TG were lower than baseline levels following lipid-lowering therapy (*p* < 0.001). Also, HDL-C concentration increased significantly after atorvastatin treatment (*p* < 0.001, Table 2).

**Table 1:** Clinical and demographic characteristics of the study group.

Parameter	*n* = 129
Age (years)	56.2 ± 10.9
Men/women (*n*)	49/80
BMI (kg/m^2^)	25.6 ± 2.7
Systolic blood pressure (mmHg)	106.8 ± 12.3
Diastolic blood pressure (mmHg)	72.7 ± 9.2
Glucose (mg/dL)	97.2 ± 9.6
TC (mg/dL)	274.9 ± 18.5
TG (mg/dL)	214.1 ± 51.9
LDL-C (mg/dL)	186.3 ± 17.4
HDL-C (mg/dL)	45.8 ± 8.3
AST/GOT (U/L)	24.4 ± 7.2
ALT/GPT (U/L)	22.7 ± 8.3
CK (U/L)	103.5 ± 77.5

Values expressed as mean ± standard deviation. *n*, number of subjects; BMI, body mass index; TC, total cholesterol; TG, triglycerides; LDL-C, low-density lipoprotein cholesterol; HDL-C, high-density lipoprotein cholesterol; AST/GOT, aspartate aminotransferase; ALT/GPT, alanine aminotransferase; CK, creatine kinase.

**Table 2:** Serum lipids levels at baseline and after treatment with atorvastatin (10 mg/day, 4 weeks).

Lipids	Baseline (mg/dL)	Treatment (mg/dL)	Change (mg/dL)	*p*-Value
TC	274.4 ± 18.3	224.5 ± 26.2	−49.8 ± 30.0	<0.001
HDL-C	46.4 ± 8.8	54.1 ± 6.7	7.6 ± 6.7	<0.001
LDL-C	185.4 ± 17.5	137.3 ± 26.1	−48.1 ± 31.6	<0.001
TG	212.8 ± 50.5	165.9 ± 48.4	−47.1 ± 43.2	<0.001

Results are expressed as mean ± SD. *p*-Values from paired *t*-test. TC, total cholesterol; HDL-C, high-density lipoprotein cholesterol; LDL-C, low-density lipoprotein cholesterol; TG, triglycerides.

### 2.2. SLCO1B1 Single Nucleotide Polymorphisms

Genotype and allelic frequencies for *SLCO1B1* SNPs are shown in Table 3. All genotype frequencies were consistent with Hardy-Weinberg equilibrium (388A>G *x*^2^ = 0.807, *p* = 0.368; 521T>C *x*^2^ = 1.064, *p* = 0.302). After 521T>C genotyping, only 1 patient was identified with the mutated 521CC genotype, while 95 patients showed the TT homozygous genotype (wild-type) and 33 individuals carried the TC genotype (heterozygous). Due to the low frequency of the CC genotype, subsequent analyses were completed clustering the TC and CC genotypes into a dominant inheritance model. In relation to the c.388A>G SNP, 69 patients were identified as heterozygous, 36 patients with the mutated 388GG homozygote genotype and 24 showed the 388AA homozygote genotype (wild-type). No significant differences were found among the genotypes identified and baseline lipid levels (P = not significant, NS).

**Table 3:** Genotype distribution and relative allele frequencies of the studied single nucleotide polymorphisms (SNPs) in *SLCO1B1* in Chilean hypercholesterolemic individuals.

SNP	Genotypes			Alleles	
rs2306283 (388A>G)	AA	AG	GG	A 0.453	G 0.547
	18.6%	53.5%	27.9%		
	(*n* = 24)	(*n* = 69)	(*n* = 36)		
rs4149056 (521T>C)	TT	TC	CC	T 0.864	C 0.136
	73.6%	25.6%	0.8%		
	(*n* = 95)	(*n* = 33)	(*n* = 1)		

### 2.3. SLCO1B1 Polymorphisms and Atorvastatin Treatment

The effect of the 388A>G and 521T>C SNPs on lipid-lowering response to 10 mg/day of atorvastatin is shown in Table 4 and Table 5, respectively. Reductions in serum TC, LDL-C and TG were not associated with the c.388A>G and c.521A>C variants (*p* > 0.05). Nevertheless, high-density lipoprotein cholesterol was significantly affected by the 388A>G polymorphism once atorvastatin treatment was completed (*p* = 0.02; Table 4). According to Tukey’s multiple comparison test, carriers of the G allele of the c.388A>G SNP displayed greater mean values of HDL-C than the wild-type AA genotype following low-dose atorvastatin medication (Table 4). In addition, the influence of the c.388A>G SNP on HDL-C levels was significant for both men (*p* = 0.039) and women (*p* = 0.049) (data not shown), demonstrating a gender-independent effect of the polymorphism.

**Table 4:** Influence of the *SLCO1B1* 388A>G SNP on atorvastatin treatment (10 mg/day, 4 weeks).

Lipids (mg/dL)	Condition	Genotypes			*p*-Value
		AA (*n* = 24)	AG (*n* = 69)	GG (*n* = 36)	
TC	Basal	275. 5 ± 20.7	275.4 ± 17.9	273.4 ± 18.5	0.81
	Treatment	228.0 ± 30.3	224.5 ± 25.9	221.6 ± 26.4	
	% Change	−17.0 ± 10.4	−18.3 ± 10.8	−18.9 ± 10.3	
HDL-C	Basal	48.9 ± 10.8	45.1 ± 7.6	44.8 ± 7.3	0.02 *
	Treatment	53.4 ± 6.7	53.6 ± 7.6	54.0 ± 4.9	
	% Change	11.6 ± 14.7 ^a^	20.0 ± 13.9 ^b^	22.5 ± 16.6 ^b^	
LDL-C	Basal	183.6 ± 21.2	187.3 ± 16.7	186.4 ± 15.9	0.27
	Treatment	139.6 ± 28.8	138.5 ± 26.8	134.1 ± 25.5	
	% Change	−21.6 ± 14.9	−25.3 ± 15.9	−28.3 ± 14.3	
TG	Basal	216.7 ± 42.5	214.8 ± 50.8	210.9 ± 60.2	0.30
	Treatment	174.2 ± 42.0	159.5 ± 49.6	164.0 ± 48.3	
	% Change	−19.3 ± 15.4	−24.9 ± 19.6	−19.8 ± 20.5	

TC, total cholesterol; HDL-C, high-density lipoprotein cholesterol; LDL-C, low-density lipoprotein cholesterol; TG, triglycerides; Data are shown as mean ± SD; *p*-Values from one-way ANOVA; Superscripts ^a^ and ^b^ obtained from multiple comparison by Tukey post-test, showing differences between genotypes AA *vs.* AG/GG; *n*, number of individuals; *, significant association.

**Table 5:** Influence of the *SLCO1B1* 521T>C SNP on atorvastatin treatment (10 mg/day, 4 weeks).

Lipids (mg/dL)	Condition	Genotypes		*p*-Value
		TT (*n* = 95)	TC + CC (*n* = 34)	
TC	Basal	273.6 ± 19.5	276.7 ± 16.0	0.58
	Treatment	223.6 ± 27.0	223.0 ± 24.9	
	% Change	−17.9 ± 10.8	−19.1 ± 10.7	
HDL-C	Basal	46.2 ± 9.2	44.3 ± 4.7	0.15
	Treatment	53.5 ± 6.6	54.0 ± 6.2	
	% Change	17.9 ± 16.1	22.4 ± 13.2	
LDL-C	Basal	185.1 ± 18.9	188.4 ± 12.8	0.34
	Treatment	137.5 ± 26.7	135.0 ± 26.0	
	% Change	−24.8 ± 16.4	−27.9 ± 14.9	
TG	Basal	211.8 ± 49.9	219.8 ± 57.0	0.92
	Treatment	162.9 ± 46.0	169.9 ± 51.1	
	% Change	−21.9 ± 18.5	−21.5 ± 20.5	

TC, total cholesterol; HDL-C, high-density lipoprotein cholesterol; LDL-C, low-density lipoprotein cholesterol; TG, triglycerides; Data are shown as mean ± SD; *p*-Values from Student’s *t*-test; *n*, number of individuals.

## 3. Discussion

Prevention efforts related to cardiovascular disease are focused primarily on the management of lipid levels, due to several clinical studies have provided sufficient evidence that alterations in lipid metabolism, specifically higher or lower levels of LDL-C and HDL-C, respectively, can increase both morbidity and mortality [16]. In this study, we explored two common variants of the *SLCO1B1* gene and their relationship with lipid-lowering response to 10 mg/day of atorvastatin therapy in hypercholesterolemic individuals from Chile. Minor allele frequencies (MAFs) observed for SNPs c.388A>G and c.521T>C were 0.54 and 0.13, respectively. A report from the highly admixed Brazilian population showed MAFs of 0.32 and 0.12 for these genetic variants [17], whilst in Chinese MAFs were 0.72 and 0.16 for both variants [18], demonstrating major ethnic differences for the c.388A>G SNP between populations. Lipid levels prior atorvastatin treatment showed no differences for the c.388A>G and c.521T>C polymorphisms. This is consistent with results documented in similar study conditions [17,18,19]. In addition, lower levels of total, LDL-C and TG, along with higher HDL-C levels (*p* < 0.001) after completion of the therapy are in accordance with a previous report obtained by our group [20]. However, despite statin effectiveness, a wide inter-individual variability was also observed. In 2013, Shabana *et al.* [21], reported no association between the c.388A>G SNP and LDL-C levels in an Egyptian cohort following atorvastatin treatment, which is similar to our results. However, they found no influence of this SNP on HDL-C concentration. Nevertheless, it must be noted that their results were obtained using a much higher atorvastatin dose (40 mg/day) on the basis of a small 50-subjects sample, which could introduce some bias to their findings, making comparisons unreliable. In fact, after 4 weeks of 40 mg/day atorvastatin therapy, they only observed a small 9.2% LDL-C reduction, which is considerably below normal reductions observed for that dose [22]. On the other hand, and under similar conditions than those applied to our study, Rodrigues *et al.* [17] described a significant LDL-C reduction in response to 10 mg/day atorvastatin therapy in homozygous individuals for the c.388G allele. However, no association was observed in relation to HDL-C, probably because they observed a reduction of this lipid fraction after statin medication instead of the usual elevation reported for HDL-C. However, this HDL-C decrease following low-dose atorvastatin found by Rodrigues is consistent with a recent pharmacogenetics study also performed on Brazilian population under similar study conditions [23], demonstrating the necessity of characterizing genetic determinants of statin response in dissimilar populations. Interestingly, when compared to the wild-type homozygote, we observed a significant impact of the mutated homozygote on HDL-C, increasing this lipid fraction as much as 10.8% after atorvastatin therapy, while heterozygous had an 8.34% increase (*p* = 0.02). Observational studies have demonstrated that HDL-C is an independent cardiovascular risk factor, and that an increase of 1 mg/dL of HDL-C leads to a reduction of 2%–3% of cardiovascular disease risk [24,25]. Furthermore, epidemiological evidence indicates that higher HDL-C levels are associated with cardioprotective effects [16]. Previously, our laboratory reported an association between the mutated genotype of *CYP3A4* (−290A>G) and *CYP3A5* (6986A>G) and increased concentrations of HDL-C [20], which may be related to our results. Our data suggest that hypercholesterolemic individuals with the homozygous mutant 388GG could be in advantage in terms of metabolic response of HDL-C compared to wild-type genotype patients treated with atorvastatin, which represent a cardiovascular protective factor in our study population.

The presence of the mutated homozygous of c.521T>C has been associated to alterations in the transport of drugs and with increased plasma levels of OATP-C in other populations [13,26]. As this genotype was present in only one individual, we could not estimate its effect. This variant did not alter the baseline lipid profile. Moreover, once atorvastatin therapy was completed, we did not observe an association between this polymorphism and lipid-lowering therapy. Our results agree with Rodrigues *et al.* where in similar conditions found no association between the c.521C allele and changes in lipid parameters after 4 weeks administration of 10 mg/day of atorvastatin [17]. Similarly, Yang *et al.* found no association between genotypes for the 521T>C variant and the therapeutic effect of pitavastatin [27]. However, it has been reported a significant relationship between the SLCO1B1*5 variant and delayed hepatocellular pravastatin uptake, with greater area under the curve (AUC) [13,14] and higher levels of simvastatin in plasma. Also, Pasanen *et al.* found that subjects with the 521CC genotype had a greater AUC of atorvastatin concentrations than those with TT genotype [28].

A major limitation for the results obtained is the restricted sample size, which also limited the observation of subjects carrying the 521CC genotype, preventing further analysis. For this reason our investigation should be interpreted in the context of its design, since the limited number of individuals included could introduce bias to the observed associations. Consequently, studies incorporating a larger cohort are needed to replicate our results.

## 4. Experimental Section

### 4.1. Subjects

A total of 129 unrelated Chilean individuals, diagnosed with hypercholesterolemia according to the National Cholesterol Education Program (NCEP) criteria [29] and treated with atorvastatin 10 mg/day for one month were studied. Patients were selected from the Federico Thieme Health Center, located in La Araucanía, Chile. None of the subjects had liver or kidney disease, endocrine disorders, diabetes, cancer, malignant diseases or were receiving concomitant lipid-lowering therapy. This investigation excluded patients with familial hypercholesterolemia, and those who were treated with diuretics and β blockers, which could affect the lipid profile. All patients signed voluntarily an informed consent. The Ethics Committee of Southern Araucanía Health Service (14 September 2007) approved the study and protocols were developed in compliance with good laboratory practice. Blood samples were obtained by venipuncture after 10 to 12 h of fasting. Biochemical measurements were determined by enzymatic methods already described [30] and LDL-C was calculated with the Friedewald equation [31]. The accuracy of biochemical measurements was ensured using normal and pathological sera.

### 4.2. Molecular Analysis

Genomic DNA was extracted from blood leukocytes by a salting out procedure optimized by Salazar *et al.* [32]. The *SLCO1B1* 521T>C polymorphism was determined by real-time polymerase chain reaction (PCR) using TaqMan allelic discrimination system (Drug Metabolism Genotyping Assays from Applied Biosystems, ID4488997, Foster City, CA, USA). PCR assays contained 12.5 μL of Universal Master Mix (2×) (Life Technologies, Carlsbad, CA, USA), 1.25 μL of TaqMan Drug Metabolism Genotyping Assay (20×, Applied Biosystems, Foster City, CA, USA) and 3 μL of DNA (20 ng) diluted in nuclease-free water. The thermal cycling protocol consisted of initial cycle at 10 min a 95 °C followed by 40 cycles at 92 °C for 10 s, 60 °C for 1 min, using standard conditions for real-time system (Life Technologies). Genotype calling was performed using the StepOne software v 2.2 (Life Technologies). For the genotyping of the c.388A>G variant, genomic DNA samples were amplified using 5′-CCCAC TATCTCAGGTGATGCTCT-3′ as the forward primer and 5′-AAACACATGCTGGGAAATTGACAGA-3′ as the reverse primer. Amplification reactions were performed in a final volume of 25 µL, containing 50 ng of genomic DNA, 160 nM of each primer, 200 μL of each dNTP, 1 U of Taq DNA polymerase, 3 mM of MgCl_2_ and PCR buffer (50 mmol/L KCl, 2 mmol/L MgCl_2_, 20 mmol/l (NH_4_)_2_SO_4_, 75 mmol/L Tris–HCl, pH 9.0). After initial denaturation at 98 °C for 3 min, the amplification was performed in 30 cycles consisting of 1 min at 94 °C, 1 min at 64 °C and 1 min at 72 °C. A final extension of 10 min at 72 °C completed the reaction. PCR amplification was performed using a PX2 Thermal Cycler of Thermo Fisher Scientific (Waltham, MA, USA). After amplification, the 266-bp PCR product was digested with Taq1 restriction enzyme (Life Technologies) in a total reaction volume of 30 µL. Wild-Type 388A/A yielded two fragments of 201 and 65 bp, and the variant G/G gave three fragment of 178, 65 and 23 bp. The heterozygotes formed four fragments of 201, 178, 65 and 23 bp. In addition, two more persons without any change reread all gels blindly, and 20% of the analyses were repeated randomly. Digestion fragments were identified by electrophoresis (2.5% agarose gel) stained with ethidium bromide and visualized using an UV transilluminator (E-Box 1000, Vilber Lourmat, Marne La Vallée, France).

### 4.3. Statistical Analysis

Statistical analysis was performed using GraphPad Prism version 5.00 for Windows (GraphPad Software, San Diego, CA, USA). Continuous variables are presented as mean ± SD. Allelic frequencies and genotype distribution were estimated by gene counting. Chi-square test (*x*^2^) was used to analyze differences in allelic frequencies and to verify Hardy-Weinberg equilibrium. Normal distribution was assessed using the Kolmogorov-Smirnov test (α = 0.05). For the comparison of the lipid profile before and after atorvastatin therapy, paired *t*-test was employed, while for comparing genotypes clustered into a dominant inheritance model Student’s *t*-test was performed. Co-dominance was evaluated by one-way ANOVA followed by Tukey’s post-hoc test, previously observing homogeneity of variance. Two-tailed *p* values <0.05 were considered as statistically significant.

## 5. Conclusions

In summary, this study demonstrates that the *SLCO1B1* rs2306283 polymorphism influences HDL-C in response to atorvastatin treatment in Chilean hypercholesterolemic subjects. No further association was found with other lipid parameters and lipid-lowering treatment. Despite the lack of association to drug therapy, which is usual in pharmacogenetic studies [33], understanding the molecular basis of this occurrence, as well as the genetic determinants that may influence the response to atorvastatin, along with the ethnic origin of the Chilean population will certainly optimize drug therapy.
